# CRISPR-Cas9 Dual-gRNA Attack Causes Mutation, Excision and Inversion of the HIV-1 Proviral DNA

**DOI:** 10.3390/v12030330

**Published:** 2020-03-18

**Authors:** Caroline S. Binda, Bep Klaver, Ben Berkhout, Atze T. Das

**Affiliations:** Laboratory of Experimental Virology, Department of Medical Microbiology, Amsterdam UMC, University of Amsterdam, 1105AZ Amsterdam, The Netherlands; carolinebinda@gmail.com (C.S.B.); g.p.klaver@amsterdamumc.nl (B.K.)

**Keywords:** HIV, CRISPR-Cas, excision, mutation, DNA repair

## Abstract

Although several studies demonstrated that the HIV proviral DNA can be effectively targeted and inactivated by the CRISPR-Cas9 system, the precise inactivation mechanism has not yet been analyzed. Whereas some studies suggested efficient proviral DNA excision upon dual-gRNA/Cas9 treatment, we previously demonstrated that hypermutation of the target sites correlated with permanent virus inactivation. To better understand the mechanism underlying HIV inactivation, we analyzed the proviral DNA upon Cas9 attack with gRNA pairs. We observed that dual-gRNA targeting resulted more frequently in target site mutation than fragment excision, while fragment inversion was rarely observed. The frequencies varied for different gRNA combinations without an obvious relationship with the distance between the target sites, indicating that other gRNA and target DNA characteristics influence the DNA cleavage and repair processes.

## 1. Introduction

**Figure 1:**
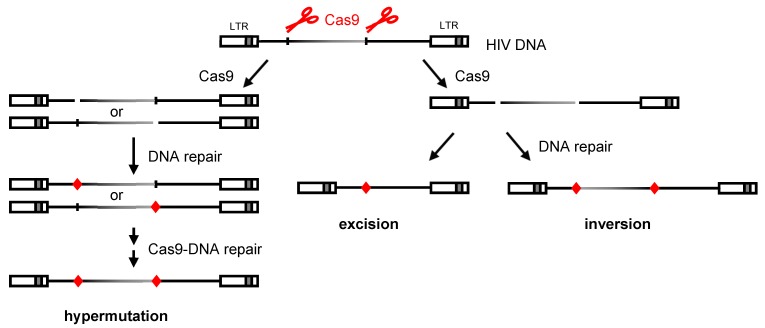
Products resulting from dual-gRNA/Cas9 targeting of proviral DNA. CRISPR-Cas9 attack of the HIV provirus with two gRNAs that target different viral domains (or with a single gRNA that targets both the 5′ and 3′ LTR domain) can result in hypermutation, excision or inversion of the viral DNA. Non-simultaneous Cas9 cleavage (left panel) will lead to the accumulation of mutations around the gRNA target regions, as the cleaved DNA will immediately be repaired by the cellular NHEJ and MMEJ repair mechanisms, which frequently introduce mutations. Simultaneous cleavage at both target sites and subsequent ligation of the free DNA ends can result in excision or inversion of the intervening fragment (right panel) (red diamond: mutation due to error-prone DNA repair).

The development of combination antiretroviral therapy (cART) has been instrumental in combating HIV infection. Although cART is capable of reducing the viral load in HIV-infected patients to undetectable levels, it does not cure the infection. One reason for this is that cART does not target the HIV proviral DNA in reservoir cells. These latently-infected cells give rise to the production of infectious virus and a viral rebound when cART is halted [1,2,3,4]. Therefore, HIV-infected individuals face lifelong therapy, and resistance to the antiviral drugs may develop. Novel antiviral strategies that aim to inactivate the viral reservoir are being investigated, including targeting of the integrated provirus with the CRISPR-Cas9 system (reviewed in [5]). Here, a guide RNA (gRNA) directs the Cas9 endonuclease to complementary sequences in the proviral DNA. Upon cleavage, the DNA is repaired by cellular DNA repair mechanisms, in particular non-homologous end-joining (NHEJ) and microhomology-mediated end-joining (MMEJ) [6,7]. This repair frequently introduces mutations at the cleavage site, mostly nucleotide deletions and insertions (indels), but also nucleotide substitutions, which can inactivate the virus. Dual-gRNA targeting of the proviral DNA may not only result in mutations clustering at both target sites, but also in excision or inversion of the intervening sequence, as previously described for the dual-gRNA/Cas9 treatment of chromosomal DNA [8,9,10,11,12] (Figure 1). Several studies aimed at the excision of large provirus fragments by using two gRNAs targeting different viral genome domains or a single gRNA targeting the LTR that is present at both the 5′ and 3′ end of HIV DNA [13,14,15,16,17,18,19,20,21]. Analysis of the proviral DNA products by PCR using primers immediately up- and downstream of the two targets suggested efficient excision [16,17,21]. However, this PCR analysis strongly favors detection of the short excision product over longer mutation and inversion products, in particular when the gRNA target sites are further apart.

Using experimental systems that support HIV replication, we and others demonstrated that HIV replication can be inhibited with Cas9 and a single antiviral gRNA. However, the virus can escape from this inhibition through the acquisition of specific mutations at the target site that prevent gRNA binding, but do not block virus replication [22,23,24,25,26]. Nucleotide substitutions were frequently observed when targeting highly conserved essential proviral sequences, whereas indels were selected when targeting less conserved regions that more easily tolerate larger mutations [22]. The position at the Cas9 cleavage site and the frequent observation of indels, which are the hallmark for NHEJ and MMEJ repair, indicate that these mutations are due to the error-prone DNA repair following Cas9 cleavage, disclosing a novel mechanism of HIV evolution [22,23,27]. The use of dual-gRNA combinations proved to be more effective at durably inhibiting viral replication [28,29]. We identified two gRNA pairs that prevented virus escape and led to complete virus inactivation [28]. Interestingly, although provirus excision could be detected, complete virus inactivation coincided with the acquisition of mutations at both target sites in the proviral DNA. Thus, hypermutation could be a major mechanism of CRISPR-Cas9-mediated HIV inactivation. We therefore set out to quantitatively asses the different outcomes of the dual-gRNA targeting of HIV proviral DNA (mutation, excision or inversion; see Figure 1) in infected cells.

## 2. Materials and Methods

### 2.1. Plasmids

**Figure 2:**
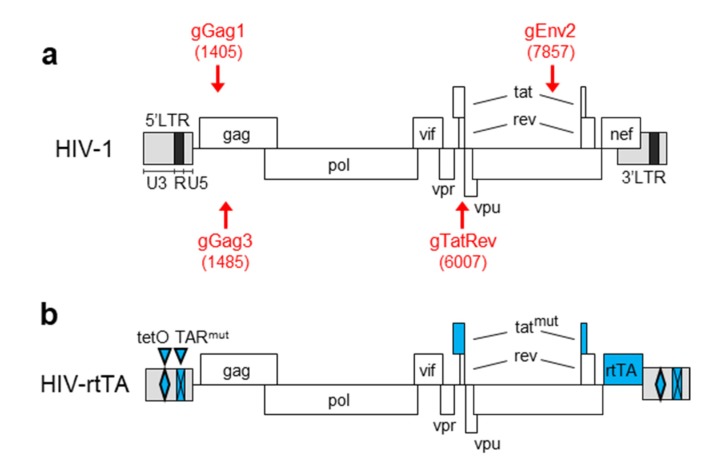
CRISPR/Cas9 targeting of HIV. (**a**) The HIV-1 proviral DNA with the position of the gRNA target sites indicated (target sequences listed in Supplementary Table S2). The gRNAs targeting the sense and antisense strand are shown above and below the DNA, respectively, with the numbers between brackets indicating the Cas9 cleavage site. (**b**) In HIV-rtTA, the TAR and Tat functions are inactivated through nucleotide substitutions, and tetO elements and the rtTA gene are inserted in the U3 domains and at the site of the nef gene, respectively. These mutations do not affect the gRNA targets.

The lentiviral vector (LV) pLenti-SpBsmBI-sgRNA-Hygro (Addgene; 62205) used for the expression of gGag1 was a gift from Rene Maehr [30]. The LVs LentiGuide-Puro (Addgene; 52963) used for the expression of gGag3, gTatRev and gEnv2 and LentiCas9-Blast (Addgene; 52962) containing the human codon-optimised *Streptococcus pyogenes* Cas9-expression cassette were gifts from Feng Zhang [31]. Construction of the gRNA-expressing vectors was previously described [22]. The plasmid pLAI encodes the HIV-1 subtype B LAI virus isolate [32]. The plasmid pHIV-rtTA encodes a doxycycline-dependent LAI derivative, in which Tat and TAR were inactivated through mutations, the rtTA_G19F-E37L-P56K-F86Y-A209T_ gene was inserted at the site of the nef gene, and two tet operator sites were inserted in the U3 promoter region (Figure 2) [33,34,35].

### 2.2. Cell Culture, Transfection and Transduction

Human embryonic kidney 293T cells and SupT1 T cells were cultured, as described previously [36]. LV particles were produced as previously described [37]. Briefly, 293T cells were transfected with the LV plasmid and packaging plasmids pSYNGP, pRSV-rev, and pVSV-g using Lipofectamine 2000. After transfection, the medium was replaced with Opti-MEM (Life Technologies), and the cells were cultured for 48 h. The LV particles-containing supernatant was centrifuged and filtered (0.45 μm). The LentiCas9-Blast particles were concentrated on a Vivaspin 20 ultrafiltration spin column (100 kDa molecular weight cut-off; Sartorius). Aliquots were stored at −80 °C. LV particle production was measured by CA-p24 antigen capture enzyme-linked immunosorbent assay (ELISA), as previously described [38]. SupT1 cells (2 × 10^5^ cells in 1 mL of culture medium) were transduced with LentiCas9-Blast particles (30 ng of CA-p24) and cultured with 3 μg/mL blasticidin for 10 days to select SupT1-Cas9 cells. To generate SupT1 cells expressing Cas9 and two gRNAs, the SupT1-Cas9 cells were first transduced with the pLenti-SpBsmBI-Hygro-gGag1 particles, followed by hygromycin B selection (500 μg/mL for 10 days), and subsequently with the LentiGuide-Puro-gGag3, gEnv2 or gTatRev particles followed by puromycin selection (0.5 μg/mL for 10 days).

### 2.3. Virus Stocks and Infection

To generate virus stocks, SupT1 cells were transfected with pLAI and pHIV-rtTA by electroporation, as described previously [39] and cultured for several days until large syncytia were produced. The virus-containing supernatant was centrifuged and filtered (0.45 μm), and the CA-p24 level was determined by ELISA. Cas9/gRNA expressing SupT1 cells (2 × 10^5^ cells in 1 mL culture medium) were infected with an equal amount of virus corresponding to 6 ng CA-p24. Virus production of HIV-rtTA was performed in the presence of 1 µg/mL doxycycline, but infection was done in the absence of doxycycline, and infected cultures were washed twice with fresh cell culture medium to remove any residual doxycycline [35].

### 2.4. PCR and qPCR Analysis

Infected cells were harvested by centrifugation, and the cellular DNA, including the proviral DNA, was isolated with the DNeasy Blood and Tissue Kit (QIAGEN) using a QIAshredder microcentrifuge spin-column (QIAGEN) to homogenize the lysate. For PCR analysis, the gRNA target region was amplified using the gGag1-forward and gGag3-reverse primers (Supplementary Table S1). The PCR product was analyzed by agarose gel electrophoresis, and the ~350–500 bp fragments were isolated from gel and cloned in a pCRII-TOPO TA cloning vector. The sequence of multiple fragments was analyzed. 

For qPCR analysis of the Cas9/gRNA-treated proviral DNA products, 5 µL cellular DNA sample was mixed with 1× LC480 MasterMix (Roche), 900 µM sense primer, 900 µM antisense primer and 200 µM 6-carboxyfluorescein (6-FAM)-labelled gGag1 probe (primer and probe sequences shown in Supplementary Table S1) in a total volume of 20 µL and analyzed with the LightCycler^®^ 480 (Roche Life Science). To produce template DNA for the qPCR standard curves, the DNA was PCR amplified with the qPCR primers and cloned in the pCRII-TOPO TA cloning vector, followed by selection of clones with the wild-type fragment or with the modified sequence resulting from excision or inversion of the region between the gRNA sites.

## 3. Results

### 3.1. PCR and Sequence Analysis of HIV Proviral DNA upon Dual gRNA/Cas9 Attack

**Figure 3:**
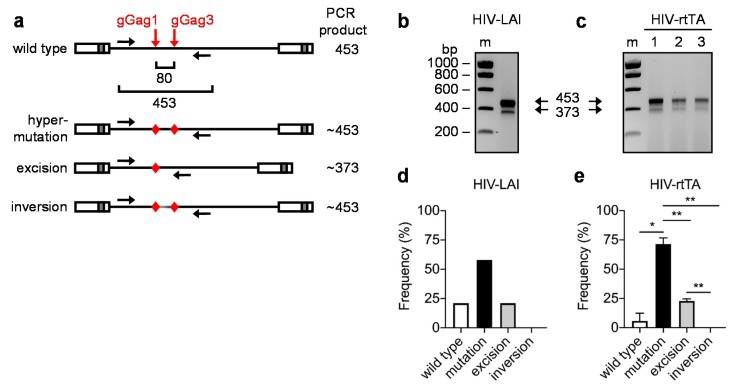
CRISPR/Cas9 targeting of HIV-1 DNA. (**a**) Polymerase chain reaction (PCR) analysis of gGag1 + gGag3 targeted proviral DNA (red arrows, gRNA target sites; black arrows, PCR primers). The distance between the Cas9 cleavage sites is 80 bp. The expected size of the PCR product is 453 bp for the wild-type product, ~453 bp for the mutation and inversion product, and ~373 bp for the excision product. (**b**,**c**) SupT1 cells stably expressing Cas9 + gGag1 + gGag3 were infected with HIV-LAI (b) and HIV-rtTA (c). Cellular DNA was isolated at 3 and 7 days post-infection, respectively. The proviral DNA was amplified by PCR and the products were analyzed by agarose gel electrophoresis. The size of the molecular weight markers (m) and the PCR products are shown. (**d**,**e**) The PCR products (~350–500 bp) were excised from the agarose gel, purified and TA cloned. Multiple clones were sequenced to determine the frequency of mutation, excision and inversion. For HIV-LAI (d; *n* = 1) the sequences are shown in Figure 3. For HIV-rtTA (e; *n* = 3), the sequences are shown in Supplementary Figure S1. To demonstrate statistically significant differences, the HIV-rtTA data were analyzed by repeated measures One-way ANOVA (* *p* < 0.05, ** *p* < 0.01; error bars indicate the SD).

PCR amplification of dual-gRNA attacked proviral DNA will favor detection of excision events when the size of the excision and non-excision (i.e., mutation and inversion) PCR products differs significantly [40,41]. To minimize such bias, we designed a dual-gRNA attack and PCR analysis strategy that results in only a small size difference between the PCR products. For this, we used gRNAs that target the viral DNA at nearby positions and PCR primers that anneal at a small distance from the target sites (Figure 3a). The gRNAs gGag1 and gGag3 target the gag region with only 80 bp between the Cas9 cleavage sites. This distance is sufficient to avoid steric hindrance when Cas9 is simultaneously recruited to both targets [42]. The PCR primers span the gGag1 + gGag3 targeted region and will result in a 453-bp amplicon for non-excised products, containing wild-type, mutated and/or inverted sequences, and in a 373-bp amplicon for the excised product (Figure 3a). As the DNA repair process that follows Cas9 cleavage frequently results in small indels at the cleavage sites, the actual size of the amplicons will vary slightly.

**Figure 4:**
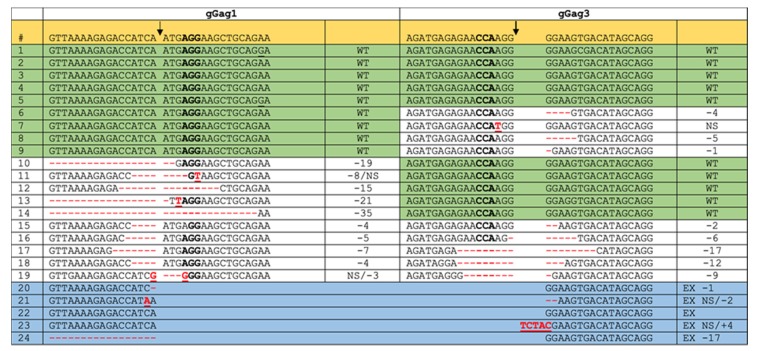
Sequence analysis of gGag1 + gGag3 targeted HIV-LAI DNA. SupT1 cells stably expressing Cas9 + gGag1 + gGag3 were infected with HIV-LAI, and cellular DNA was extracted at 3 days post-infection (as described in Figure 3). The gRNA target regions were amplified by PCR, the PCR products were TA cloned and 24 clones were sequenced. Sequences were aligned to the wild-type reference HIV-LAI sequence (highlighted in yellow; PAM sequence in bold) and 17 nucleotides on either side of the Cas9 cleavage sites (indicated by black arrows) are shown. Wild-type (WT; green), mutation (white) and excision (EX; blue) products are shown (mutations in red; NS, nucleotide substitution; -n, n nucleotides deleted; +n, n nucleotides inserted).

SupT1 cells were stably transduced with Cas9-, gGag1- and gGag3-expressing lentiviral vectors and infected with the HIV-1 LAI strain (HIV-LAI). The integrated proviral DNA was isolated at 3 days post-infection, and the gGag1 + gGag3 region was amplified by PCR. The PCR product was first visualized on an agarose gel (Figure 3b), which showed two bands with the expected sizes of ~453 and ~373 bp. The PCR product was cloned into a TA cloning vector, and 24 clones were sequenced (Figure 4). Five of these clones carried the unedited, wild-type sequence at both target sites, five were mutated at the gGag1 site only, four at the gGag3 site only, and five at both target sites. Most mutations were small indels, which is typical for the NHEJ and MMEJ DNA repair that follows Cas9 cleavage. In five of the cloned fragments, the region between the two target sites had been excised, while we did not observe any inversion events. This dual-gRNA attack thus resulted more frequently in the mutation of at least one target (in total 14 of the 24 sequences; 58%) than excision of the viral DNA fragment (5/24; 21%) (Figure 3d).

The clones with a wild-type HIV sequence (5/24; 21%) either represent uncleaved viral DNA or Cas9-cleaved DNA that was perfectly repaired. As previously shown [28], extended culturing can increase the level of DNA cleavage and reduce the wild-type frequency, but may lead to the selection of virus variants that escape from dual-gRNA inhibition. Such escape was indeed observed for the gGag1 + gGag3 combination [28]. To avoid this problem, we used a doxycycline-controlled HIV variant to analyze the Cas9 induced mutational pattern at later time points. In this HIV-rtTA strain, the Tat-TAR transcription activation mechanism is inactivated and functionally replaced by the doxycycline-inducible Tet-On transcription mechanism [33,35,39] (Figure 2).

Three parallel SupT1 cell cultures stably expressing Cas9, gGag1 and gGag3 were infected with HIV-rtTA in the absence of doxycycline, which does allow virus infection and integration of the viral DNA, but prevents subsequent rounds of viral replication. The integrated proviral DNA was analyzed by PCR at 7 days post-infection, thus allowing Cas9 to act for a week. For all three independent HIV-rtTA cultures, the larger PCR product of ~453 bp was more abundant than the smaller ~373 bp product (Figure 3c). TA cloning and sequencing of the PCR products demonstrated that the average level of wild-type fragments was only 6% (Supplementary Figure S1: 1/23, 3/23 and 0/24 clones in cultures 1–3), while one or both gRNA targets were mutated in 71% of the fragments (17/23, 15/23 and 18/24 clones, respectively), and the intervening region was excised in 23% of the HIV genomes (5/23, 5/23 and 6/24 clones, respectively) (Figure 3e). The intervening region was never found to be inverted. In summary, dual gRNA/Cas9 attack of HIV DNA with gGag1 and gGag3, which bind to nearby positions in the proviral genome, leads more frequently to mutation than excision, while the inversion frequency was too low to be detected.

### 3.2. Quantitative PCR Analysis of HIV Proviral DNA upon Dual gRNA/Cas9 Attack

**Figure 5:**
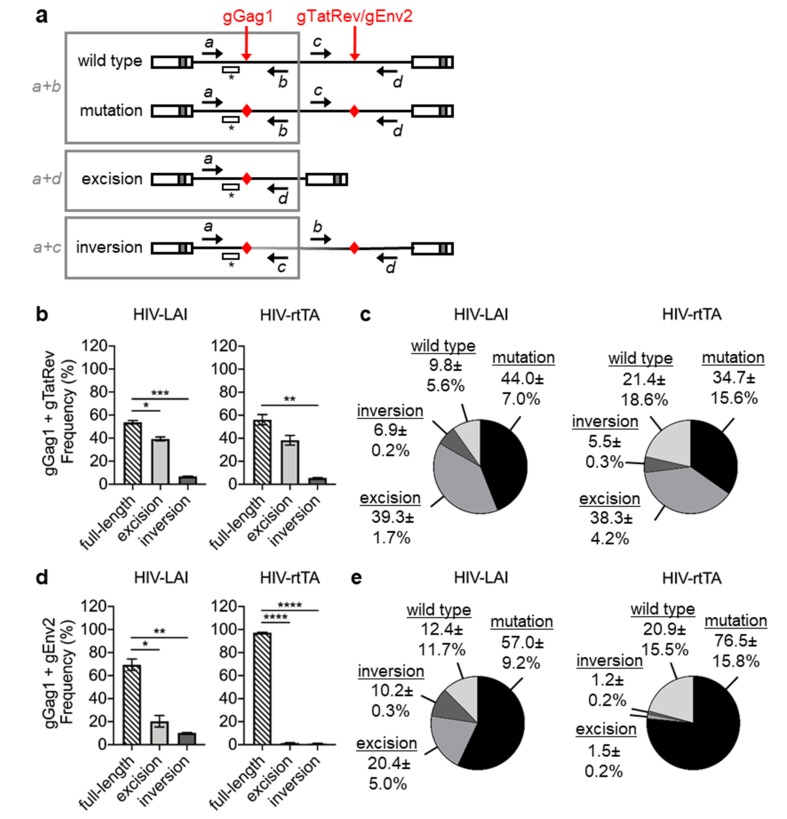
Quantitative PCR analysis of gGag1 + gTatRev and gGag1 + gEnv2 targeted proviral DNA products. (**a**) Schematic of the HIV-1 DNA with the position of the gRNA target sites indicated by red arrows, the position of qPCR primers *a − d* indicated by black arrows, and the target site of the FAM-labelled qPCR probe indicated by *. Primer combination *a + b* detects full-length fragments with either a wild-type or mutated sequence, *a + d* detects excision products and *a + c* detects inversion products. (**b**–**e**) SupT1 cells stably expressing Cas9 and gGag1 + gTatRev (**b**,**c**) or gGag1 + gEnv2 (**d**,**e**) were infected with HIV-LAI and HIV-rtTA and cellular DNA was isolated at 7 days post-infection. The full-length, excision and inversion products were quantitated by qPCR with the different primer combinations and the frequency of each product is shown in b and d (sum of all products set at 100%). The full-length PCR product (primers *a + b*) was TA cloned and 16–24 clones were sequenced to determine the ratio between wild-type and mutated fragments (sequences shown in Supplementary Figure S2 for gGag1 + gTatRev and Supplementary Figure S3 for gGag1 + gEnv2). This ratio was combined with the qPCR data to calculate the frequency of the wild-type, mutation, excision and inversion products shown in c and e. To demonstrate statistically significant differences, the qPCR data were analyzed by repeated measures One-way ANOVA (*n* = 3; * *p* < 0.05; ** *p* < 0.01, *** *p* < 0.001, **** *p* < 0.0001; error bars indicate the SD).

To analyze the proviral DNA modifications upon dual-gRNA/Cas9 attack with considerable distance between the gRNA target sites, we selected two gRNA pairs that were previously shown to durably inhibit HIV-1 replication [28]. These gRNA pairs target the gag region and either the overlapping tat/rev region (gGag1 + gTatRev; 4602 bp between the targets) or the env region (gGag1 + gEnv2; 6452 bp) (Figure 2a). Because PCR analysis with up- and downstream primers, as used for the gGag1 + gGag3 analysis, would result in preferential amplification of the excision product and the underestimation of other products (wild-type, mutation and inversion), we developed quantitative PCRs for the full-length viral DNA (primers *a + b* in Figure 5a), the excision product (primers *a + d*) and the inversion product (primers *a + c*). As the full-length fragments (primers *a + b*) can have either a wild-type or mutated gGag1 site, this PCR product was TA cloned and sequenced to determine the ratio between these outcomes.

SupT1 cells stably expressing Cas9, gGag1 and either gTatRev or gEnv2 were infected with HIV-LAI or HIV-rtTA. At 7 days after infection, the integrated proviral DNA in three cultures was analyzed with the different qPCRs to determine the ratio between the full-length, excision and inversion products. Quantification of the different HIV-LAI DNA products in gGag1 + gTatRev cells showed that 54% of the proviral DNA was full-length, whereas 39% corresponded to the excision product and 7% to the inversion product (Figure 5b). Sequence analysis demonstrated that the gGag1 site was mutated in most of the full-length fragments (in 17/23, 15/16 and 10/13 of the sequences analyzed for the different cultures; Supplementary Figure S2a–c). The combined data show that mutation at the gGag1 site had occurred in 44% of the proviruses, excision in 39% and inversion in 7%, whereas 10% of gGag1 targets were unedited (Figure 5c). Proviral DNA analysis of the HIV-rtTA-infected gGag1 + gTatRev cultures (qPCR in Figure 5b; full-length product sequencing in Supplementary Figure S2d–f) yielded comparable frequencies: 35% mutation, 38% excision, 6% inversion and 21% wild type (Figure 5c). We thus measured a similar frequency of the mutation and excision of the proviral DNA upon gGag1 + gTatRev targeting for both HIV-LAI (44% and 39%, respectively) and HIV-rtTA (35% and 38%, respectively). In fact, the mutation frequency is likely to be slightly higher, as proviruses with an unedited gGag1 site (10% and 21% for HIV-LAI and HIV-rtTA, respectively) may have been mutated at the gTatRev site. 

The qPCR analysis of the HIV-LAI and HIV-rtTA infected gGag1 + gEnv2 cultures showed the presence of significantly more full-length proviral DNA compared to the excision and inversion products (Figure 5d). Subsequent sequence analysis of TA-cloned fragments revealed that the majority of the full-length DNA was mutated at the gGag1 site (82% for HIV-LAI and 79% for HIV-rtTA; Supplementary Figure S3). The combined data indicate that mutation had occurred in 57% of the HIV-LAI and 77% of the HIV-rtTA DNA, whereas excision and inversion of the intervening fragment were observed at much lower frequencies (respectively 20% and 10% for HIV-LAI and 2% and 1% for HIV-rtTA) (Figure 5e).

In summary, dual gRNA/Cas9 attack of HIV DNA with the gGag1 + gTatRev and gGag1 + gEnv2 pairs, with considerable distance between the target sites, did result in a similar frequency of mutation and excision of the proviral DNA or to the more frequent mutation than excision of the proviral DNA, respectively, while the inversion frequency was low for both pairs.

## 4. Discussion

Several studies demonstrated that HIV can be targeted and inactivated by the CRISPR-Cas9 system (reviewed in [5]). Both excision and hypermutation of the proviral DNA were suggested as the major route to inactivation, but the analyses were incomplete and biased toward detection of the excision product. To better understand the mechanism underlying HIV inactivation, we analyzed the proviral DNA upon dual-gRNA/Cas9 attack using PCR/sequencing and qPCR methods that are less biased by the size difference between the excised and mutated products. We here show that dual-gRNA targeting can result in target site mutation, fragment excision and fragment inversion, but at varying efficiencies.

Three gRNA pairs with different spacing between the target sites were tested. For this small sample set, the frequency of mutation, excision and inversion could not be linked to the distance between target sites. Mutations were more frequently observed than excisions for the gGag1 + gGag3 and gGag1 + gEnv2 pairs with target sites located about 80 bp and 6.5 kbp apart, respectively, while the mutation frequency was similar to the excision frequency for the gGag1 + gTatRev combination with 4.6 kbp between the target sites. Inversions were detected at a low frequency in the gGag1 + gEnv2 and gGag1 + gTatRev treated proviral DNA, which were analyzed by qPCR, and not at all in the gGag1 + gGag3 treated DNA, which was analyzed by sequencing. However, this latter lack of detection was likely due to the fact that only a limited number of proviral DNA fragments were sequenced.

Although target site mutations will often block virus replication (e.g., indels that cause a frameshift in an open reading frame), specific mutations may be selected that allow the virus to escape (e.g., nucleotide substitutions and 3-nucleotide indels that do not disturb the reading frame) [22,23,27]. We previously demonstrated such HIV escape, but also that viral escape can be prevented by the simultaneous targeting of two highly conserved and essential viral domains [28]. It is important to realize that even if virus replication is blocked, the expression of viral proteins may continue. Such HIV protein-expressing cells can be eliminated by cytotoxic T cells [43,44,45] but may contribute to increased levels of immune activation and inflammation [46,47]. Therefore, excision of the near-complete proviral DNA, or a large proviral DNA fragment, may be the preferred outcome of dual-gRNA/Cas9 treatment, as this would not only block virus escape but also prevent viral protein expression [13,14,15,16,17,18,19,20,21].

Excision will require Cas9 cleavage of both targets, followed by ligation of the DNA ends, which should occur before the DNA repair of one of the lesions takes place (Figure 1). Once either target site is mutated, further cleavage at this position may be prevented, thus avoiding additional rounds of CRISPR attack that could establish excision of the proviral DNA. Because of the quick cellular DNA repair process, differences in cleavage kinetics at the Cas9 targets may stimulate the generation of mutations at the expense of excision. All tested gRNA pairs included gGag1, and the observed variation in the excision and mutation frequencies may indicate different cleavage kinetics for the second gRNA (gGag3, gTatRev and gEnv2), which could be due to differences in gRNA or target site characteristics. The relatively high excision frequency of gGag1 + gTatRev could be facilitated by more similar Cas9 cleavage kinetics. Indeed, when analysing the capacity of the gGag1 + gTatRev pair to mutate integrated proviral DNA [28], gGag1 and gTatRev seemed to have similar activity, with 38% of the gGag1 and 25% of the gTatRev targets remaining wild type after 12 days of culture. In contrast, when testing the gGag1 + gEnv pair, gEnv2 seemed to be much more effective than gGag1 as no wild-type gEnv2 target sites were observed, while again 38% of the gGag1 sites remained wild type. The latter difference in Cas9 activity with gGag1 and gEnv2 may also explain the modest excision frequency observed with this gRNA pair. However, our gRNA set is small, and we did not measure other factors, like the intracellular concentration of the different gRNAs. The similar-kinetics requirement may be more easily fulfilled with a single gRNA targeting the identical sequence in the 5’ and 3’ LTR, although the chromosomal environment will differ, which could influence the cleavage and repair processes. Future studies focusing on the parameters that impact the mutational outcome of dual-gRNA attack, e.g., characteristics of the gRNA or the target site, will be important to identify gRNA pairs that lead to maximal excision.

We here focused on the mechanism of virus inactivation in T cells that are harnessed with dual-gRNAs and Cas9 using optimized in-vitro lentiviral transduction and selection conditions. Translation of this antiviral strategy into a cure for HIV-infected persons remains a significant challenge. A sterilizing cure would require that all HIV-infected cells are targeted with the gRNA and Cas9 components, but reducing the viral load below a certain level may suffice (functional cure). Several delivery methods are available, e.g., for transient editing with gRNA-Cas9 ribonucleoprotein particles or virus-like particles [48,49,50], and for durable editing with adeno-associated virus or lentiviral vectors [17,21,51], but these methods may not reach sufficient infected cells in vivo. Novel delivery methods may be needed that specifically target HIV-reservoir cells, possibly facilitated by a specific marker like CD32a [52,53]. Other prominent issues relate to off-target effects due to Cas9 cleavage at non-target sites in the cellular DNA and immune responses against the non-human Cas9 protein [54,55]. The high genetic diversity of HIV can be another complicating factor, as even single nucleotide mismatches between the gRNA and target sequence can prevent Cas9 recognition [56]. The simultaneous use of multiple gRNAs that target highly conserved sequences seems the best strategy to circumvent this problem, although exotic HIV isolates with divergent target sequences may require specific adaptation of the gRNA molecules [56,57,58,59].

## Supplementary Materials

The following are available online at https://www.mdpi.com/1999-4915/12/3/330/s1, Figure S1: Sequence analysis of gGag1 + gGag3 targeted HIV-rtTA DNA, Figure S2: Sequence analysis of gGag1 + gTatRev targeted proviral DNA, Figure S3: Sequence analysis of gGag1 + gEnv2 targeted proviral DNA, Table S1: Primers and probe used for PCR and qPCR analysis, Table S2: Selected gRNAs targeting HIV-1.
