# Supplementary material for: CRISPR-Cas9 Dual-gRNA Attack Causes Mutation, Excision and Inversion of the HIV-1 Proviral DNA
Source: Viruses. 2020 Mar 18;12(3):330. doi: 10.3390/v12030330 (PMC7150824; doi:10.3390/v12030330)
Supplement: Supplementary file 1 [file viruses-12-00330-s001.pdf]

a

I	gGag1			gGag3		
#	GTTAAAAGAGACCATCA	ATGAGGAAGCTGCAGAA		AGATGAGAGAACCAAGG	GGAAGTGACATAGCAGG	
1	GTTAAAAGAGACCATCA	ATGAGGAAGCTGCAGAA	WT	AGATGAGAGAACCAAGG	GGAAGTGACATAGCAGG	WT
2	GCTAAAAGAGACCATCA	ATGAGGAAGCTGCAGAA	WT	AGATGAGAGTACCAAGG	--AAGTGACATAGCAGG	-2
3	GTTAAAAGAGACCATC-	ATGAGGAAGCTGCAGAA	-1	AGATGAGAGAACCAAGG	GGAAGTGACATAGCAGG	WT
4	GTTAAAAGAGACCATCAC	ATGAGGAAGCTGCAGAA	+1	AGATGAGAGAACCAAGG	GGAAGTGACATAGCAGG	WT
5	GTTAAAAGAGACCA---	-TGAGGAAGCTGCAGAA	-4	AGATGAGAGAACCAAGG	GGAAGTGACATAGCAGG	WT
6	GTTAAAAGAG-------	------AAGCTGCAGAA	-13	AGATGAGAGAACCAAGG	GGAAGTGACATAGCAGG	WT
7	GTTAAAAGAGACCATCA	ATGGGGAAGCTGCAGAA	NS	AGATGAGAGAACCAAGG	GGAAGTGACATAGCAGG	WT
8	GTTAAAAGAGAGGA---	-------AGCTGCAGAA	-10	AGATGAGAGAACCAAGG	GGAAGTGACATAGCAGG	WT
9	GTTAAAAGAGACCA---	----------GGCAGAA	-13/NS	AGATGAGAGAACCAAGG	-GAAGTGACATAGCAGG	-1
10	GTTAAAAGAG-------	-----GAAGCTGCAGAA	-12	AGATGAGAGAACCAAGG	--AAGTGACATAGCAGG	-2
11	GTTAAAAGAGACCATC-	-TGAGGAAGCTGCAGAA	-2	AGATGAGAGAACCAAGG	--GTGTGACATAGCAGG	-2/NS
12	GTTAAAAGAGACCA---	--------------GAG	-17/NS	AGATGAGAGAACCAAGG	--GAGTGACATAGCAGG	-2/NS
13	GTTAAAAGAGACCATC-	-----------------	-50	AGATGAGAGAACCAAGG	C----------------	NS/-23
14	GTTAAAAGAGACCTA--	--GAGGAAGCTGCAGAA	NS/-4	-----------------	---AGTGACATAGCAGG	-24
15	GTTAAAAGAGACCAGGCC	ATGAGGAAGCTGCAGAA	NS/+1	AGATGAGAG--------	-----------------	-44
16	GTTAAA-----------	--GAGGAAGCTGCAGAA	-13	-----------------	GGAAGTGACATAGCAGG	-28
17	GTTAAAAGAGACCATCA	-TGAGGAAGCTGCAGAA	-1	AGATGAGAGAACCAAGG	GCGAAGTGACATAGCAGG	+1
18	GTTAAAAGAGACCACT-	--GAGGAAGCTGCAGAA	-3	AGATGAGAGAACCAAGG	-GAAGTGACATAGCAGG	-1
19	GTTAAAAGAGACCATCA				GGAAGTGACATAGCAGG	EX
20	GTTAAAAGAGACCATCA				GGAAGTGACATAGCAGG	EX
21	-----------------				-----------------	EX -156
22	GTTAAAAGAGACC----				-----------AGCAGG	EX -15
23	GTTAAAAGAGGAAGCTTAACA				GGAAGTGACATAGCAGG	EX +4

b

II	gGag1			gGag3		
#	GTTAAAAGAGACCATCA	ATGAGGAAGCTGCAGAA		AGATGAGAGAACCAAGG	GGAAGTGACATAGCAGG	
1	GTTAAAAGAGACCATCA	ATGAGGAAGCTGCAGAA	WT	AGATGAGAGAACCAAGG	GGAAGTGACATAGCAGG	WT
2	GTTAAAAGGGACCATCA	ATGAGGAAGCTGCAGAA	WT	AGATGAGAGAACCAAGG	GGAAGTGACATAGCAGG	WT
3	GTTAAAAGAGACCATCA	ATGAGGAAGCTGCAGAA	WT	AGATGAGAGAACCAAGG	GGAAGTGACATAGCAGG	WT
4	GTTAAAAGAGACCATCA	ATGAGGAAGCTGCAGAA	WT	AGA--------------	-----------------	-31
5	GGTAA------------	------AAGCTGCAGAA	-18	AGATGAGAGAACCAAGG	GGAAGTGACATAGCAGG	WT
6	GTTAAAAGAGACCA---	ATGAGGAAGCTGCAGAA	-3	AGATGAGAGAACCAAGG	GGAAGTGACATAGCAGG	WT
7	GTTAAAAGAGACCAT--	------------CAGAA	-14	AGATGAGAGAACCAAGG	GGAAGTGACATAGCAGG	WT
8	GTTAAAAGA--------	--GAGGAAGCTGCAGAA	-10	AGATGAGAGAACCAAGG	--AAGTGACATAGCAGG	-2
9	GTTAAAAGAGACC----	ATGAGGAAGCTGCAGAA	-4	-----------------	----GGGACATAGCAGG	-36
10	GTTAAAAGAGACCATCA	-----------------	-17	AGATGAGAGAACCAAGG	--AAGTGACATAGCAGG	-2
11	GTT--------------	--TAGGAAGCTGCAGAA	-16/NS	AGG--------------	GGAAGTGACATAGCAGG	NS/-14
12	GTTAAAAGAGACCATATGTTACTA	ATGAGGAAGCTGCAGAA	NS/+7	AGATGAGAGAACCAAGG	--AAGTGACATAGCAGG	-2
13	GTTAAAAGAGACCATCC	ATGAGGAAGCTGCAGAA	NS	AGATGAGAGAAC-----	-----------------	-45
14	-----------------	--------GCTGCAGAA	-31	AGATGAGAGAACCAAGG	GATCGAAGGGGACATAGCAGG	+4/NS
15	-----------------	------AAGCTGCAGAA	-73	AGATGAGAGAACCAAGG	-------------CAGG	-13
16	GTTAAAA----------	-----GAAGCTGCAGAA	-15	AGATGAGAGAACCAAG-	-----TGACATAGCAGG	-6
17	GTTAAAAGAGACCATTAGG	ATGAGGAAGCTGCAGAA	NS/+2	AGATGAGAGAACCAAGG	GCGGAAGTGACATAGCAGG	+2
18	GTTAAAAGAGACCATCA	------------CAGAA	-12	AGATGAGAGAACCAAGG	GTGGAAGTGACATAGCAGG	+2
19	GTTAAAAGAGACCATC-				GGAAGTGACATAGCAGG	EX -1
20	GTTAAAAGAGACCATCA				GGAAGTGACATAGCAG	EX
21	GTTAAAAGAGACTA---				-----------AGCAGG	EX -14
22	GTTAAAAGAGACCATCA				GGAAGTGACATAGCAGG	EX
23	GTTAAAAGAGACCATCA				---------------GG	EX -15

c

III	gGag1			gGag3		
#	GTTAAAAGAGACCATCA	ATGAGGAAGCTGCAGAA		AGATGAGAGAACCAAGG	GGAAGTGACATAGCAGG	
1	GTTAAAAGAGACCAT--	--GAGGAAGCTGCAGAA	-4	AGATGAGAGAACCAAGG	GGAAGTGACATAGCAGG	WT
2	GTTAAAAGAGACCCA--	ATGAGGAAGCTGCAGAA	NS/-2	AGATGAGAGAACCAAGG	GGAAGTGACATAGCAGG	WT
3	GTTA-------------	---------------GC	-28/NS	AGATGAGAGAACCAAGG	GGAAGTGACATAGCAGG	WT
4	GTTAAAAGAG-------	-----GAAGCTGCAGAA	-11	AGATGAGAGAACCAAGG	GGAAGTGACATAGCAGG	WT
5	CA---------------	ATGAGGAAGCTGCAGAA	NS/-15	AGATGAGAGAACCAAGG	GGAAGTGACATAGCAGG	WT
6	GTTAAAAGAGACCATC-	ATGAGGAAGCTGCAGAA	-1	AGATGAGAGAACCAAGG	GGAAGTGACATAGCAGG	WT
7	GTTAAAAGAGACCCA--	ATGAGGAAGCTGCAGAA	NS/-2	AGATGAGAGAACCAAGG	GGAAGTGACATAGCAGG	WT
8	GTTAAAAGAGACC----	--------------GAA	-18	AGATGAGAGAACCAAGG	GGAAGTGACATAGCAGG	WT
9	G----------------	------------AGGAA	-28/NS	AGATGTGAGAACCAAGG	GGAAGTGACATAGCAGG	WT
10	-----------------	-----------------	-36	AGATGAGAGAACCAAGG	GGAAGTGACATAGCAGG	WT
11	GTTAAAAGAGACC----	ATGAGGAAGCTGCAGAA	-4	AGATGAGAGAACCAAGG	-AAAGTGACATAGCAGG	-1/NS
12	GTTAAAAGAGACCCC--	-TGAGGAAGCTGCAGAA	NS/-3	AGATGAGAGAACCAAGG	-----TGACATAGCAGG	-5
13	GTTAAAAGGCA------	ATGAGGAAGCTGCAGAA	-6	-----------------	--------------ATG	-32
14	GTTAAAAGAGACCATC-	-------AGCTGCAGAA	-8	AGATGAGAGAACCAAGG	-----GAGCATAGCAGG	-5/NS
15	GTTAAAAGAGACCAT--	-----------------	-20	AGATGAGAGAACCAAGG	AAGGAAGTGACATAGCAGG	+2
16	GTTAAAAGA--------	--GAGGAAGCTGCAGAA	-10	AGATGAGAGAAAAC---	--------CATAGCAGG	NS/-11
17	-----------------	-----------------	-38	AGATGAGAGAACCAAGG	-GAAGTGACATAGCAGG	-1
18	GTTAA------------	------AAGGTGCAGAA	-18	AGATGAGAGAACCAAGG	--AAGTGACATAGCAGG	-2
19	G----------------				-----------GAGCAG	EX -27/NS
20	GTTAAAAGAGACCATC-				----------GAGCAGG	EX -11/NS
21	GTTAAAAGAGACCATCA				GGAAGTGACATAGCAGG	EX
22	GTTAAAAGAGACCATCA				GGAAGTGACATAGCAGG	EX
23	GTTAAAAGAGACCATCA				GGAAGTGACATAGCAGG	EX
24	GTTAAAAGAGACCATC-				GGAAGTGACATAGCAGG	EX -1

Figure S1. Sequence analysis of gGag1+gGag3 targeted HIV-rtTA DNA. SupT1 cells stably expressing Cas9+gGag1+gGag3 were infected with HIV-rtTA and cellular DNA was isolated at 7 days post-infection as described in Figure 3 (n=3; **a-c**). The gRNA target regions were amplified by PCR, the PCR products were TA cloned and 23-24 cloned fragments were sequenced. Sequences were aligned to the wild-type reference HIV-LAI sequence (highlighted in yellow; PAM sequence in bold) and 17 nucleotides on either side of the Cas9 cleavage sites (indicated by black arrows) are shown. Wild-type (WT; green), mutation (white) and excision (EX; blue) products are indicated (mutations in red; NS, nucleotide substitution; -n, n nucleotides deleted; +n, n nucleotides inserted).

a HIV-LAI

I	gGag1		
#	GTTAAAAGAGACCATCA	ATGAGGAAGCTGCAGAA	
1	GTTAAAAGAGACCATCA	ATGAGGAAGCTGCAGAA	WT
2	GTTAAAAGAGACCATCA	ATGAGGAAGCTGCAGAA	WT
3	GTTAAAAGAGACCATCA	ATGAGGAAGCTGCAGAA	WT
4	GTTAAAAGAGACCATCA	ATGAGGAAGCTGCAGAA	WT
5	GTTAAAAGAGACCATCA	ATGAGGAAGCTGCAGAA	WT
6	GTTAAAAGAGACCATCA	ATGAGGAAGCTGCAGAA	WT
7	GTTAAAA----------	--GAGGAAGCTGCAGAA	-12
8	GTTAAAAGAGACCATC-	ATGAGGAAGCTGCAGAA	-1
9	GTTA-------------	-----------------	-57
10	GTTAAAAGAGACCATCAC	CTGAGGAAGCTGCAGAA	+1/NS
11	GTTAAAAGAGACAGAAT	GGGAGGAAGCTGCAGAA	NS
12	GTTAAAAGA--------	-----GAAGCTGCAGAA	-13
13	GT---------------	---------CTGCAGAA	-24
14	GTTAAAAGAGA------	---------CTGCAGAA	-15
15	GTTCCAAGAGACCGTT-	ATAAGGAAGCTGCAGAA	NS/-1
16	GTTAAAAGAGACCTCAT	----CGAAGCTGCAGAA	NS/-4
17	GTTAA------------	ATGAGGAAGCTGCAGAA	-12
18	GTTAAAAGAGACCATC-	-----------GCAGAA	-11
19	GTTA-------------	-------------AGAG	-26/NS
20	GTTAAAAGAGACCTTCT	ATGAGGAAGCTGCAGAA	NS
21	GTTAAAA----------	--GAGGAAGCTGCAGAA	-12
22	GTTAAAAGAGACC----	ATGAGGAAGCTGCAGAA	-14
23	GTTAAAAG---------	-----------------	-44

b

II	gGag1		
#	GTTAAAAGAGACCATCA	ATGAGGAAGCTGCAGAA	
1	GTTAAAAGAGACCATCA	ATGAGGAAGCTGCGGAA	WT
2	GTTAAAAGAGACCATC-	ATGAGGAAGCTGCAGAA	-1
3	GTTAAAAGAGACC----	ATGAGGAAGCTGCAGAA	-4
4	GTTAAAAGAGAC-----	---------CTGCAGAA	-14
5	GTTAAA-----------	---AGGAAGCTGCAGAA	-14
6	GTTAAAAGAGACCATC-	ATGAGGAAGCTGCAGTA	-1
7	GTTAAAAGAGACCATCAGA	ATGAGGAAGCTGCAGAA	+2
8	GTTAAAAGAGACCATC-	ATGAGGAAGCTGCAGAA	-1
9	GTTAAAAGAGACC----	ATGAGGAAGCTGCAGAA	-4
10	GTTAAAAGAGACCATCA	-----CCAGCTGCAGAA	-5/NS
11	-----------------	-----------------	-40
12	GTTAAAAGAGACCATCAA	ATGAGGAAGCTGCAGAA	+1
13	-----------------	----GGAAGCTGCAGAA	-22
14	GTTAAAAGAGACA----	ATGAGGAAGCTGCAGAA	-4
15	GTTAAAAGAGACA----	ATGAGGAAGCTGCAGAA	-4
16	GTTAAAAGAGACCATCAA	ATGAGGAAGCTGCAGAA	+1

c

III	gGag1		
#	GTTAAAAGAGACCATCA	ATGAGGAAGCTGCAGAA	
1	GTTAAAAGAGACCATCA	ATGAGGAAGCTGCAGAA	WT
2	GTTAAAAGAGACCATCA	ATGAGGAAGCTGCAGAA	WT
3	GTTAAAAGAGACCATCA	ATGAGGAAGCTGCAGAA	WT
4	GTTAAAAGAGACGAA--	ATGAGGAAGCTGCAGAA	NS/-2
5	GTTAAAAGAGACCA---	ATGAGGAAGCTGCAGAA	-3
6	GCTAAAAGAGACCATCACCCT	ATGAGGAAGCTGCAGAA	+4
7	GTTAAAAGAGACCATCTT	ATGAGGAAGCTGCAGAA	NS/+1
8	GTTA-------------	------------CAGAA	-19
9	GTTAAAAGAGACA----	ATGAGGAAGCTGCAGAA	-4
10	-----------------	-----------------	-79
11	GTTAAAAGAGAC-----	---------CTGCAGAA	-14
12	GTTAAAAGAGACCATC-	ATGAGGAAGCTGCAGAA	-1
13	GTTAAAAGAGACCATCAA	ATGAGGAAGCTGCAGAA	+1

d HIV-rtTA

I	gGag1		
#	GTTAAAAGAGACCATCA	ATGAGGAAGCTGCAGAA	
1	GTTAAAAGAGACCATCA	ATGAGGAAGCTGCAGAA	WT
2	GTTAAAAGAGACCATCA	ATGAGGAAGCTGCAGAA	WT
3	GTTAAAAGAGACCATCA	ATGAGGAAGCTGCAGAA	WT
4	GTTAAAAGAGACCATCA	ATGAGGAAGCTGCAGAA	WT
5	GTTAAAAGAGACCATCA	ATGAGGAAGCTGCAGAA	WT
6	GTTAAAAGAGACCATCA	ATGAGGAAGCTGCAGAA	WT
7	GTTAAAAGAGACCATCA	ATGAGGAAGCTGCAGAA	WT
8	GTTAGAAGAGACCATCA	ATGAGGAAGCTGCAGAA	WT
9	GTTAAAAGAGACCATCA	ATGAGGAAGCTGCAGAA	WT
10	GTTAAAAGAGACCATCA	ATGAGGAAGCTGCAGAA	WT
11	GTTAAAAGAGACCATCA	ATGAGGAAGCTGCAGAA	WT
12	GTTAAAAGAGACCATCA	ATGAGGAAGCTGCAGAA	WT
13	GTTAAAAGAGACCATCA	ATGAGGAAGCTGCAGAA	WT
14	GTTAAAAGAGACCATCA	ATGAGGAAGCTGCAGAA	WT
15	GTTAAAAGAGACCATCA	ATGAGGAAGCTGCAGAA	WT
16	GTTAAAAGAGACCATCA	ATGAGGAAGCTGCAGAA	WT
17	GTTAAAAGAGACCATCA	ATGAGGAAGCTGCAGAA	WT
18	-----------------	-----------------	-67
19	-----------------	--------GCTGCAGAA	-26
20	GTTAAAAGAGACCATC-	ATGAGGAAGCTGCAGAA	-1
21	GTTAAAAGAGACCATCAACC	ATGAGGAAGCTGCAGAA	+3
22	GTTAAAAGAG-------	-----------------	-34
23	GTTAAAAGAGACCATCC	ATGAGGAAGCTGCAGGA	NS
24	GTTAAAAGAGACCATCA	GTGAGGAAGCTGCAGAA	NS

e

II	gGag1		
#	GTTAAAAGAGACCATCA	ATGAGGAAGCTGCAGAA	
1	GTTAAAAGAGACCATCA	ATGAGGAAGCTGCAGAA	WT
2	GTTAAAAGAGACCATCA	ATGAGGAAGCTGCAGAA	WT
3	GTTAAAAG---------	-------AGCTGCAGAA	-16
4	GTTAAAAGAGACCATC-	------GAGCTGCAGAA	-7/NS
5	GTTAAAAGAGACCAT--	ATGAGGAAGCTGCAGAA	-2
6	GTTAAAAGAGACC----	ATGAGGAAGCTGCAGAA	-4
7	GTTAAAAGAGACCATCAA	ATGAGGAAGCTGCAGAA	+1
8	GTTAAAAGAGACC----	ATGAGGAAGCTGCAGAA	-4
9	GTTAAAAGAGACCATC-	ATGAGGAAGCTGCAGAA	-1
10	GTTAAAAGAGACCA---	-----------GCAGAA	-14
11	G----------------	-----------------	-42
12	GTTAAAAGAGACCATCAA	ATGAGGAAGCTGCAGAA	+1
13	GTTAAAAG---------	-------AGCTGCAGAA	-16

f

III	gGag1		
#	GTTAAAAGAGACCATCA	ATGAGGAAGCTGCAGAA	
1	GTTAAAAGAGACCATCA	ATGAGGAAGCTGCAGAA	WT
2	GTTAAAAGAGACCATCA	ATGAGGAAGCTGCAGAA	WT
3	GTTAAAAGAGACCATCA	ATGAGGAAGCTGCAGAA	WT
4	GTTAAAAGAGACCATCA	ATGAGGAAGCTGCAGAA	WT
5	GTTAAAAGAGACCATC-	ATGAGGAAGCTGCAGAA	-1
6	GTTAAAAGAGACCATCAA	ATGAGGAAGCTGCAGAA	+1
7	GTTAAAAGAGACCATCAA	ATGAGGAAGCTGCAGAA	+1
8	GTTAAAAGAGACCATCAC	ATGAGGAAGCTGCAGAA	+1
9	GTTAAAAGAGACCATC-	ATGGGGAAGCTGCAGAA	-1/NS
10	GTTAAAAGAGACCATCAA	ATGAGGAAGCTGCAGAA	+1
11	GTTAAAAGAGACCATCAA	ATGAGGAAGCTGCAGAA	+1
12	GTTAAAAGAGACC----	ATGAGGAAGCTGCAGAA	-4
13	GTTAAAAGAGACCAT--	CTGAGGAAGCTGCAGAA	-2/NS
14	-----------------	-----------------	-149
15	-----------------	-----------------	-76
16	-----------------	-----GAAGTTGCAGAA	-111/NS

Figure S2. Sequence analysis of gGag1+gTatRev targeted proviral DNA. SupT1 cells stably expressing Cas9+gGag1+gTatRev were infected with HIV-LAI (n=3; **a-c**) or HIV-rtTA (n=3; **d-f**) and cellular DNA was isolated at 7 days post-infection as described in Figure 5. The gGag1 target region was amplified through PCR, PCR products were TA cloned and 13-24 clones were sequenced. Sequences are shown as described in Supplementary Figure S1.

a HIV-LAI

I	gGag1		
#	GTTAAAAGAGACCATCA	↓ATG**AGG**AAGCTGCAGAA	
1	GTTAAAAGAGACCATCA	ATG**AGG**AAGCTGCAGAA	WT
2	GTTAAAAGAGACCATCA	ATG**AGG**AAGCTGCAGAA	WT
3	GTTAAAAGAGACCATCA	ATG**AGG**AAGCTGCAGAA	WT
4	GTTAAAAGAGACCATCA	ATG**AGG**AAGCTGCAGAA	WT
5	GTTAAAAGAGACCATCA	ATG**AGG**AAGCTGCAGAA	WT
6	GTTAAAAGAGACCATCA	ATG**AGG**AAGCTGCAGAA	WT
7	GTTAAAAGAGACCATCA	ATG**AGG**AAGCTGCAGAA	WT
8	GTTAAAAGAGACCATCA	ATG**AGG**AAGCTGCAGAA	WT
9	GTTAAAAGAGACCATCA	-----**G**AAGCTGCAGAA	-5
10	-----------------	--G**AGG**AAGCTGCAGAA	-22
11	GTTAAAAGAGAC-----	----**TT**AAGCTGCAGAA	-9/NS
12	GTTAAA-----------	---**AGG**AAGCTGCAGAA	-14
13	GTTAAAAGAGAC**A**----	ATG**AGG**AAGCTGCAGAA	NS/-4
14	-----------------	-----------------	-80
15	GTTAAAAGAGACCATCA**A**	ATG**AGG**AAGCTGCAGAA	+1
16	GTTA-------------	-----------GCAGAA	-24
17	GTTAAAAGAGACC----	ATG**AGG**AAGCTGCAGAA	-4
18	GTTAAAAGAGACCATCA**A**	ATG**AGG**AAGCTGCAGAA	+1
19	GTTAAAAGAGACCAT--	---**AGG**AAGCTGCAGAA	-5
20	GTTAAAAGAGACCATCA**GA**	ATG**AGG**AAGCTGCAGAA	+2
21	GTTAAAAGAGACC----	----**GG**AAGCTGCAGAA	-8
22	GTTAAAAGAGACC----	ATG**AGG**AAGCTGCAGAA	-4
23	GTTAAAAGAGAC-----	ATG**AGG**AAGCTGCAGAA	-5

b

II	gGag1		
#	GTTAAAAGAGACCATCA	↓ATG**AGG**AAGCTGCAGAA	
1	GTTAAAAGAGACCATCA	ATG**AGG**AAGCTGCAGAA	WT
2	GTAAAAAGAGACCATCA	ATG**AGG**AAGCTGCAGAA	WT
3	GTTAAAAGAGACCATCA	ATG**AGG**AAGCTGCAGAA	WT
4	GTTAAAAGAGACCATCA	--G**AGG**AAGCTGCAGAA	-2
5	GTTAAAAGAGACCA---	ATG**AGG**AAGCTGCAGAA	-3
6	GTTAAAAGAGAC**A**----	ATG**AGG**AAGCTGCAGAA	NS/-4
7	GTTAAAAGAGACC----	ATG**AGG**AAGCTGCAGAA	-4
8	GTTAAAAGAGACCATC-	ATG**AGG**AAGCTGCAGAA	-1
9	GTTAAAAGAGACCATCA**A**	ATG**AGG**AAGCTGCAGAA	+1
10	GTTAAAAGAGACCATCA	----------**A**GCAGAA	-10/NS
11	GTTAAAAGAGACCATCA	-----------------	-17
12	GTTAAAAGAGACCATCA	-----**T**AAGCTGCAGAA	-5/NS
13	GTTAAAAGAGACCATCA**A**	ATG**AGG**AAGCTGCAGAA	+1
14	GTTAAA-----------	---**AGG**AAGCTGCAGAA	-14
15	GTCAAAAGAGACCATC-	ATG**AGG**AAGCTGCAGAA	-1
16	GTTAAAAGAGACCATCA**A**	ATG**AGG**AAGCTGCAGAA	+1

c

III	gGag1		
#	GTTAAAAGAGACCATCA	↓ATG**AGG**AAGCTGCAGAA	
1	GTTAAAAGAGACCATC-	ATG**AGG**AAGCTGCAGAA	-1
2	GTTAAAAGAGAC-----	----**TC**AAGCTGCAGAA	-9/NS
3	GTTAAAAGAGACCATC**CCATT**	**C**TG**AGG**AAGCTGCAGAA	NS/+4
4	GTTAAAAGAGACCATC-	---**AGG**AAGCTGCAGAA	-4
5	G----------------	-----------------	-35
6	GTTAAAAGA--------	ATG**AGG**AAGCTGCAGAA	-8
7	GTTAAAAGAGACCATCA**GG**	ATG**AGG**AAGCTGCAGAA	+2
8	GTTAAAAGAGACC----	ATG**AGG**AAGCTGCAGAA	-4
9	-----------------	ATG**AGG**AAGCTGCAGAA	-19
10	GTTA-------------	---------CGACAGAA	-22
11	GTTAAAAGAGACCA---	-----**GG**AGCTGCAGAA	-8/NS
12	GTT--------------	---------CTGCAGAA	-23
13	GTTAAAAGAGA------	-------AGCTGCAGAA	-13
14	-----------------	-----------------	-42
15	-----------------	ATG**AGG**AAGCTGCAGAA	-33

d HIV-rtTA

I	gGag1		
#	GTTAAAAGAGACCATCA	↓ATG**AGG**AAGCTGCAGAA	
1	GTTAGAAGAGACCATCA	ATG**AGG**GAGCTGCAGAA	WT
2	GTTAAAAGAGACCATCA	ATG**AGG**AAGCTGCAGAA	WT
3	GTTAAAAGAGACCATCA	ATG**AGG**AAGCTGCAGAA	WT
4	GTTAAAAGAGACCATCA	ATG**AGG**AAGCTGCAGAA	WT
5	GTTAAAAGAGACCATCA	ATG**AGG**AAGCTGCAGAA	WT
6	GTTAAAAGAGACCATCA	ATG**AGG**AAGCTGCAGAA	WT
7	GTTAAAAGAGACCATCA	ATG**AGG**AAGCTGCAGAA	WT
8	GTTAAAAGAGACCATCA	ATG**AGG**AAGCTGCAGAA	WT
9	GTTAAAAGAGACCATCA**A**	ATG**AGG**AAGCTGCAGAA	+1
10	GTTAAAAGAGAC-----	------AAGCTGCAGAA	-11
11	GTTAAAAGAGACCATC**TTTTAAC**	ATG**AGG**AAGCTGCAGAA	NS/+6
12	GTTAAAAGAGACCATCA**A**	ATG**AGG**AAGCTGCAGAA	+1
13	GTTAAAAGAGACCATCA**GA**	ATG**AGG**AAGCTGCAGAA	+2
14	GTTAAAAGAGAC-----	---------CTGCAGAA	-14
15	GTTAAAAGAG-------	------**TG**GCTGCAGAA	-13/NS
16	GTTAAA-----------	---**AGCGG**GCTGCAGAA	-14/NS
17	GTTAAAAGAGACC**T**---	**C**TG**AGG**AAGCTGCAGAA	NS/-3
18	GTTAAAAGAGACCATC-	ATG**AGG**AAGCTGCAGAA	-1
19	GTTAAAA----------	--G**AGG**GAGCTGCAGAA	-12
20	GTTAAAAGAGAC-----	ATG**AGG**AAGCTGCAGAA	-5
21	-----------------	--------GC**C**GCAGAA	-25/NS

e

II	gGag1		
#	GTTAAAAGAGACCATCA	↓ATG**AGG**AAGCTGCAGAA	
1	GTTAAAAGAGACCATCA	ATG**AGG**AAGCTGCAGAA	WT
2	GTTAAAAGAGACCATCA**A**	ATG**AGG**AAGCTGCAGAA	+1
3	GTTAAAAGAGACCATCA**TTTGT**	ATG**AGG**AAGCTGCAGAA	+5
4	GTTAAGA----------	ATG**AGG**AAGCTGCAGAA	-10
5	-----------------	-----------------	-129
6	GTTAAAAGAGACC----	ATG**AGG**AAGCTGCAGAA	-4
7	G----------------	----**GG**AAGCTGCAGAA	-20
8	GTTAAAAGA--------	--G**AGG**AAGCTGCAGAA	-10
9	GTTAAAAGAGACCATCA**TTTGT**	ATG**AGG**AAGCTGCAGAA	+5
10	GTTAAA-----------	---**AGG**AAGCTGCAGAA	-14
11	GTTAAAAGAGACCATC-	ATG**AGG**AAGCTGCAGAA	-1
12	GTTAAAAGAGACCATCA**AA**	ATG**AGG**AAGCTGCAGAA	+2
13	-----------------	-----------------	-79
14	GTTAAAAGAGACCATC-	ATG**AGG**AAGCTGCAGAA	-1
15	GTTAAAAGAGACCATCA**A**	ATG**AGG**AAGCTGCAGAA	+1
16	GTTAAA-----------	---**AGG**AAGCTGCAGAA	-14

f

III	gGag1		
#	GTTAAAAGAGACCATCA	↓ATG**AGG**AAGCTGCAGAA	
1	GTTAAAAGAGACCATCA	ATG**AGG**AAGCTGCAGAA	WT
2	GTTAAAAGAGACCATCA	ATG**AGG**AAGCTGCAGAA	WT
3	GTTAAAAGAGACCATCA	ATG**AGG**AAGCTGCAGAA	WT
4	GTTAAAAGAGACCATCA**A**	ATG**AGG**AAGCTGCAGAA	+1
5	GTTAAAAGAGACCATCA**GA**	ATG**AGG**AAGCTGCAGAA	+2
6	GTTAAA-----------	---**AGG**AAGCTGCAGAA	-14
7	GTTAAAAGAGACCATCA**A**	ATG**AGG**AAGCTGCAGAA	+1
8	GTTAAAAGAGAC-----	--**AG**GGAAGCTGCAGAA	-7/NS
9	GTTAAA-----------	---**AGG**AAGCTGCAGAA	-14
10	GTTAAAAGAGACCATCA**A**	ATG**AGG**AAGCTGCAGAA	+1
11	GTTAAAAGAGACCAT--	-----------------	-20
12	GTTAA------------	---**AGG**AAGCTGCAGAA	-15
13	GTTAAAAGAGACCA---	--**A**AGGAAGCTGCAGAA	-5/NS
14	GTTAAAAGAGA**A**-----	A**G**G**AGG**AAGCTGCAGAA	NS/-5
15	GTTAAAAGAGACCATCA**A**	ATG**AGG**AAGCTGCAGAA	+1

Figure S3. Sequence analysis of gGag1+gEnv2 targeted proviral DNA. SupT1 cells stably expressing Cas9+gGag1+gEnv2 were infected with HIV-LAI (n=3; **A-C**) or HIV-rtTA (n=3; **D-F**) and cellular DNA was isolated at 7 days post-infection (as described in Figure 4). The gGag1 target region was amplified through PCR, PCR products were TA cloned and 15-23 clones were sequenced. Sequences are shown as described in Supplementary Figure S1.

Table S1. Primers and probe used for PCR and qPCR analysis

name	sequence
gGag1-forward (a)	ACCTAGAACTTTAAATGCATGG
gGag3-reverse (b)	CGGTCTACATAGTCTCTAAAGG
gTatRev-forward (c)	GAGCCAGTAGATCCTAGAC
gTatRev-reverse (d)	CTACTACTAATGCTGCTATTGC
gEnv2-forward (c)	GCAAAGAGAAGAGTGGTG
gEnv2-reverse (d)	AGCCAGGATTCTTGCC
gGag1-probe	CCCATGTTTTCAGCATTATCAGAAGGAGCC

Table S2. Selected gRNAs targeting HIV-1

Name	Target Sequence + PAM (underlined)	Orientation
gGag1	GTTAAAAGAGACCATCAATGAGG	Sense
gGag3	CCAAGGGGAAGTGACATAGCAGG	Antisense
gTatRev	CCTATGGCAGGAAGAAGCGGAGA	Antisense
gEnv2	GGAGCAGCAGGAAGCACTATGGG	Sense
